# Mapping Contributions of the Anterior Temporal Semantic Hub to the Processing of Abstract and Concrete Verbs

**DOI:** 10.1002/hbm.70210

**Published:** 2025-04-24

**Authors:** Emiko J. Muraki, Penny M. Pexman, Richard J. Binney

**Affiliations:** ^1^ Department of Psychology University of Calgary Calgary Canada; ^2^ Hotchkiss Brain Institute University of Calgary Calgary Canada; ^3^ Department of Psychology Western University London Canada; ^4^ Cognitive Neuroscience Institute School of Psychology and Sport Science, Bangor University Bangor Wales UK

**Keywords:** abstract concepts, distortion‐corrected fMRI, embodied cognition, semantic representation, verb knowledge

## Abstract

Multiple representation theories of semantic processing propose that word meaning is supported by simulated sensorimotor experience in modality‐specific neural regions, as well as in cognitive systems that involve processing of linguistic, emotional, and introspective information. According to the hub‐and‐spoke model of semantic memory, activity from these distributed cortical areas feeds into a primary semantic hub located in the ventral anterior temporal lobe (vATL). In the present pre‐registered study, we examined whether different types of abstract verbs (mental, emotional and nonembodied) and concrete (embodied) verbs all engage the vATL, and also whether they differentially recruit a broader set of distributed neurocognitive systems (consistent with multiple representation theories). Finally, we investigated whether there is information about different verb types distributed across the broader ATL region, consistent with a Graded Semantic Hub Hypothesis. We collected data from 30 participants who completed a syntactic classification task (is it a verb? Yes or no) and a numerical judgment task which served as an active but less semantic baseline task. Whole brain univariate analyses revealed consistent BOLD signal throughout the canonical semantic network, including the left inferior frontal gyrus, left middle temporal gyrus, and the vATL. All types of abstract verbs engaged the vATL except for mental state verbs. Finally, a multivariate pattern analysis revealed clusters within the ATL that were differentially engaged when processing each type of abstract verb. Our findings extend previous research and suggest that the hub‐and‐spoke hypothesis and the graded semantic hub hypothesis provide a neurobiologically constrained model of semantics that can account for abstract verb representation and processing.

## Graphical abstract

We tested hypotheses derived from the hub‐and‐spoke model and multiple representation theories of semantic memory, providing novel evidence that (1) the ventral anterior temporal lobe (ATL) is engaged in processing abstract verbs, and (2) there are graded contributions across the ATL for different kinds of semantic information.

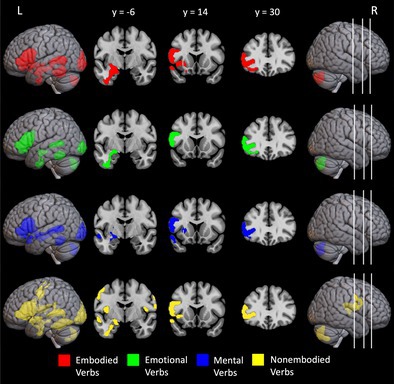

## 1 Introduction

Language plays a major role in how we interact with the world around us, yet we lack a complete cognitive and neural explanation for how we represent the meaning of words and phrases. A key unanswered question is how we understand abstract words, that is, words that cannot be directly experienced through the senses (e.g., *idea* or *regret*, as contrasted against concrete words, like *apple* or *kick*). Multiple representation theories of concept knowledge propose that word meaning is grounded in sensory, motor, emotional, and other introspective experiences, as well as linguistic representations (for an overview see Meteyard et al. 2012; Muraki et al. 2023). This occurs across a distributed semantic network that includes modality‐specific and multimodal associative brain regions (Binder and Desai 2011; Tong et al. 2022), and these theories are able to account for both concrete and abstract concepts. Some also make predictions about their brain basis. One highly influential example is the hub‐and‐spoke account, which offers a neurobiologically constrained multiple representation model of semantic memory, supported by a wealth of multi‐method evidence (Lambon Ralph et al. 2017). According to this theory, semantic processing involves (i) a distributed set of modality‐specific brain regions (i.e., the spokes) and (ii) a supramodal semantic hub which is located in the bilateral anterior temporal lobes (ATL; Binney et al. 2010; Lambon Ralph et al. 2017; Patterson et al. 2007; Patterson and Lambon Ralph 2016). The purpose of the present study is to examine (1) if the ATL is engaged as a semantic hub for processing abstract verbs and (2) whether different abstract word types differentially engage the semantic network, both at the level of cortical and subcortical “spoke” regions, as well as within the ATL hub.

## 2 The ATL Semantic Hub

A defining feature of the hub‐and‐spoke account is the proposal that the bilateral ATLs play a centrally important role as a supramodal hub for semantic processing. The ATL hub serves two main functions: (1) it mediates transmodal interactions between different types of input from the “spokes,” and (2) it integrates multimodal information across time and contexts resulting in the distillation of coherent, generalizable concepts (Lambon Ralph 2014; Lambon Ralph et al. 2010). These functions may be particularly important for abstract concepts whose meaning is more heavily dependent on linguistic and socioemotional experience and emerges from exposure over diverse contexts (Dove et al. 2022; Hoffman et al. 2013; Pexman et al. 2023). Until recently, however, there was a paucity of functional neuroimaging evidence regarding the ATL hub. This is largely due to severe and localized signal loss and image distortion that occurs with standard fMRI acquisitions (Devlin et al. 2000). However, the development of ATL‐optimized fMRI techniques has paved the way for a series of neuroimaging studies that demonstrate pervasive ATL activity in response to a range of meaning‐imbued stimuli, including pictures, environmental sounds, and both concrete and abstract word stimuli (Hoffman et al. 2015; Visser, Embleton, et al. 2010; Visser and Lambon Ralph 2011). This is further bolstered by direct intracranial recordings, surface electrophysiology, and neuromodulation studies of neurotypical adults (Binney et al. 2010, 2012, 2016; Binney and Lambon Ralph 2015; Chen et al. 2013, 2016; Marinkovic et al. 2003; Pobric et al. 2007; Rahimi et al. 2022; Rice et al. 2018).

Methodological developments have also facilitated more detailed accounts of the topology of ATL function, including the graded semantic hub hypothesis (Bajada et al. 2017; Binney et al. 2012; Jackson et al. 2018; Patterson and Lambon Ralph 2016; Rice et al. 2015). According to a graded hub view, a large and bilateral volume of anterior temporal cortex (inclusive of the temporal poles and anterior portions of all temporal gyri) comprises a unified representational space, all of which is engaged by semantic processing. However, there exists a center‐point of this hub, located within the ventrolateral ATL (vATL; inclusive of the anterior fusiform and inferior temporal gyri), which is engaged by semantic information of any kind and is invariant to, for example, the modality through which concepts are accessed. Away from the center and towards the edges of the broader ATL region, there are gradual shifts in semantic function such that regions on the periphery are relatively more specialized (Bajada et al. 2019; Binney et al. 2012; Rice et al. 2015), and encode certain types of semantic features, such as those primarily experienced through particular sensorimotor modalities (e.g., vision or audition; for a computational exploration of this general hypothesis, see Plaut 2002). As we shall explore in more detail in the next section, the graded hub hypothesis can account for a growing number of observations of differential engagement of ATL subregions for concepts of different types, including abstract and social concepts (Binney 2016; Hoffman et al. 2015).

## 3 The Nature and Neural Basis of Abstract Concepts

Contemporary accounts of abstract concept representation propose that (i) information beyond sensorimotor and verbal experience contributes to the meanings of concepts, (ii) that concreteness is a continuum rather than a dichotomy, and (iii) that there is greater heterogeneity than previously thought, such that there may be different types of abstract (and concrete) concepts (Banks and Connell 2023; Barsalou et al. 2018; Kiefer and Harpaintner 2020). Behavioral evidence has shown that both abstract and concrete concepts can be strongly associated with sensorimotor experience (Banks and Connell 2023), interoception (i.e., sensations inside the body; Connell et al. 2018), and social experience (Diveica et al. 2024a, 2024b). There is also convergent neural evidence for these claims. Systematic reviews and meta‐analyses have found that emotion concepts engage the bilateral amygdala, and in some cases the orbitofrontal cortex (Conca et al. 2021; Desai et al. 2018; Kuhnke et al. 2023) and mental state concepts are associated with activity in parts of the theory of mind network (e.g., medial prefrontal cortices and the temporo‐parietal junction, Conca et al. 2021; Desai et al. 2018). Unfortunately, these studies were restricted to noun processing (Conca et al. 2021) or included sentences and story‐level stimuli (Desai et al. 2018), such that there remains little understanding of the way in which some concepts, like individual verbs, are processed (Zwaan 2014). Nonetheless, these studies suggest, overall, that word meaning may be grounded not only in sensorimotor and linguistic experience, but also via emotional, social, and other mental experiences.

The hub‐and‐spoke account can accommodate multiple sources of semantic information (or “spokes”), but its central premise is that integration of this information takes place in the ATL hub, and this is irrespective of the nature or type of concept. There is neuroimaging evidence that demonstrates equivalent engagement of the hub's vATL center‐point for both abstract and concrete concepts (Binney et al. 2016; Hoffman et al. 2015; Rice et al. 2018). Other studies have shown differences in the ATL, with abstract concepts more strongly engaging the dorsolateral ATL than concrete concepts (Binder et al. 2009; Bucur and Papagno 2021; Wang et al. 2010). Most fMRI studies of abstract concepts, however, have not been ATL‐optimized and therefore, there is still much to be gleaned about the way different subregions are engaged by different kinds of concepts.

Different types of abstract concepts engage different ATL subregions in a way that is consistent with the graded semantic hub hypothesis, although once again these studies focus primarily on nouns. Social and emotional abstract concepts have been associated with dorsal polar regions of the ATL (Binney et al. 2016; Conca et al. 2021; Desai et al. 2018; Kuhnke et al. 2023; Wang et al. 2019) and mental state abstract concepts have been associated with various ATL subregions, including the middle and inferior temporal gyri (Desai et al. 2018). Furthermore, differential patterns of activation for word meanings with visual versus auditory features have been observed in the anterior middle and inferior temporal gyri (Murphy et al. 2017). According to the graded semantic hub hypothesis, differential engagement of ATL subregions to certain types of semantic information reflects preferential connectivity to inputs from sensorimotor, linguistic, and other pre‐semantic processes (Binney et al. 2012). The sensitivity of dorsal/polar subregions to socioemotional features may, for example, reflect dense connectivity to frontal limbic regions (via the uncinate fasciculus; Bajada et al. 2019; Papinutto et al. 2016). On the other hand, the vATL center‐point is equally connected to all inputs and therefore should respond equally to all kinds of concepts. It has yet to be fully and systematically explored, however, whether (i) the vATL is engaged by all the above concept types (while using appropriate ATL‐optimized methods), (ii) the differential engagement reflects abstractness more generally, and (iii) similar differences arise for verbs.

## 4 The Present Study: Abstract Verbs

Abstract verbs are understudied, despite the fact that they feature frequently in our vocabulary and can convey important information about changes in mental states and other circumstances that are not directly observable, such as socioemotional dynamics. This paucity contrasts with extensive (including brain‐based) investigations comparing different types of nouns (e.g., concrete and abstract) and exploring action (concrete) verb processing (Li et al. 2022; Muraki et al. 2020). In addition, there is ample evidence from patient data and imaging studies with healthy adults to suggest that nouns and verbs involve different representations, either due to their grammatical class or to differences in the semantic content that varies systematically between the grammatical classes, so we cannot assume that findings related to abstract noun processing generalize to abstract verbs (Beber et al. 2019; Cappa and Perani 2003; Elli et al. 2019; Lukic et al. 2021; Vigliocco et al. 2011). A small number of fMRI studies have investigated specific types of abstract verbs. These have shown that abstract emotion verb processing is associated with activity in the left temporal pole and middle temporal gyrus, as well as widespread activity through the motor and somatosensory cortices when compared to baseline conditions or motion verbs (Moseley et al. 2012; Rodriguez‐Ferreiro et al. 2011). This suggests that both emotional and motor experiences contribute to processing these types of abstract verbs. Mental state (or cognition) verbs have been associated with activity in the left posterolateral temporal cortex when compared to motion verbs (Grossman et al. 2002). Further, change‐of‐state verb processing has been associated with activity in the ventrotemporal cortex (Kemmerer et al. 2008), a region also involved with motion perception. These studies did not compare different abstract verb types, nor did they closely examine the role of the ATL hub.

More recently, Muraki et al. (2020) used electroencephalogram (EEG) activity to investigate the neural correlates of processing emotion verbs, mental state verbs, and nonembodied abstract verbs (i.e., verbs not related to experiences of the human body, including change‐of‐state verbs such as *dissolve* or *evaporate*). Nonembodied abstract verbs had a significantly more negative N400 amplitude compared to the mental state verbs, detected at frontal electrodes. The N400 is an event‐related potential component that has been associated with integrating new information during semantic processing. Furthermore, consistent with the aforementioned fMRI studies, a distributed source analysis identified the temporal pole and inferior temporal cortex as more associated with processing mental state abstract verbs, whereas processing nonembodied abstract verbs was associated with posterior ventrotemporal cortex. Therefore, there is preliminary evidence to suggest at least partially distinct network supporting different verb types.

In the present study, we aimed to identify neural correlates of processing different types of abstract and embodied (or concrete) verbs and, in doing so, to test the predictions of multiple representation theories of semantic processing and the graded hub‐and‐spoke account. We extended the study of Muraki et al. (2020) using distortion‐corrected fMRI to reveal the involvement of the distributed cortical and subcortical “spoke” regions, and the ATL semantic hub. We used the same syntactic classification task (SCT) as Muraki et al. (2020; Muraki et al. 2022), because it has reliably identified semantic effects in behavioral and EEG studies on verbs (Muraki et al. 2020; Muraki et al. 2022; Sidhu et al. 2014). This is likely because grammatical classes are proposed to arise in part from pragmatic/semantic meaning (Vigliocco et al. 2011), therefore semantic dimensions should contribute to grammatical class decisions, particularly in languages for which grammatical information is not embedded in the morphology of non‐inflected verbs (Frau et al. 2025). Our pre‐registered hypotheses were as follows. If multiple representation theories are correct, then different verb types will differentially recruit a distributed set of cortical and subcortical regions (outside the ATL). If the hub‐and‐spoke account is correct, then all verb types will engage the ATL semantic hub. Third, if the graded semantic hub hypothesis is correct, the vATL will be engaged by all verb types, whereas other ATL subregions may show differential patterns of activation associated with particular types of abstract verbs.

## 5 Materials and Method

### 5.1 Preregistration and Open Practices Statement

This study (hypotheses, method, and analysis plans) was pre‐registered via the Open Science Framework (available at: https://osf.io/j9kub). Our stimuli are available for download at the Open Science Framework project for the current study, as are the resulting statistical maps from our analyses (https://osf.io/vd9ba/).

### 5.2 Design Considerations

The approach taken in the present study was optimized to be sensitive to Blood‐Oxygen Level‐Dependent (BOLD) signal across all parts of the ATL, given this brain region is especially prone to magnetic susceptibility‐induced signal loss and image distortion in echo planar imaging (EPI) (Devlin et al. 2000; Visser, Jefferies, et al. 2010). We used the same acquisition parameters and distortion correction procedures as Balgova et al. (2022), which obtained a good signal to noise ratio in the ATL. We used a dual‐echo gradient‐echo EPI fMRI sequence, which acquires images at both a short echo time (12 ms) that is less prone to signal loss due to spin dephasing and a long echo time (35 ms) that is more sensitive to BOLD contrast. This dual‐echo sequence is more effective at detecting signal in inferior temporal regions compared to standard single gradient‐echo sequences and spin‐echo sequences (Halai et al. 2014, 2015). Moreover, we acquired the images with a left‐to‐right phase‐encoding direction, which reduces signal pileup in the inferior ATL (Balgova et al. 2022; Embleton et al. 2010). Finally, we applied a post‐acquisition k‐space spatial correction procedure to address geometric distortions in the data, which provides a more effective method of distortion correction compared to other commonly used procedures, including those based on B0 field maps (Embleton et al. 2010).

### 5.3 Participants

Thirty participants took part in the experiment (21 female, 8 male, 1 nonbinary, mean age = 22.7, SD age = 4.25). All participants were between the ages of 18–40, first‐language English speakers, with normal or corrected‐to‐normal vision, no history of neurological or psychiatric conditions (based on self‐report), and were assessed to be right‐handed using the Edinburgh Handedness Inventory (Oldfield 1971). Two participants were excluded from data analysis due to poor task performance (they did not have significantly greater than chance accuracy on either the SCT or the numerical judgment task (NJT) as per our exclusion criteria in the preregistration), leaving 28 participants remaining in the final analysis (19 female, 8 male, 1 nonbinary, mean age = 22.39, SD age = 3.46). The study was conducted at Bangor University and was approved by the local research ethics review committee. Participants received monetary compensation in exchange for study participation.

### 5.4 Experimental and Baseline Tasks

Participants completed a two‐alternative forced‐choice SCT, which was selected because the task decision is broad enough to involve dimensions of meaning that are important to verbs generally, without biasing participants to attend to a specific dimension of meaning which may be relevant to only one type of verb (e.g., *is it an action or nonaction*; Pexman et al. 2017). On each trial participants were presented with a written word in the middle of a computer screen and asked to decide if the word was a verb, to which they responded either yes or no. We did not mention nouns in our framing of the task decision, as prior research has shown that this can attenuate semantic effects related to verb processing (Muraki et al. 2022). Rather, the task was framed to focus on categorization of verb or not. The baseline task was a two‐alternative forced‐choice (NJT). On each trial participants were presented with a number in the middle of a computer screen and asked to decide if the number was odd (or alternatively, asked to decide if a number was even), to which they responded either yes or no. The NJT task decision was counterbalanced across participants, such that half the participants made an “odd” judgment and half made an “even” judgment. The NJT was selected as a baseline task to provide a comparable visual stimulus (same font size and a similar range of length in digits for the numbers and letters for the words) and decision type (two‐alternative forced choice) to that of the SCT, and with the assumption that it would require similar levels of attention, but minimal semantic processing (unlike a lexical decision task, which would provide more comparable visual stimuli, but is likely to also elicit some semantic processing). The NJT has been successfully employed as an active baseline in prior studies examining semantic processing in the ATL (Binney et al. 2010, 2016).

### 5.5 Stimuli

#### 5.5.1 Words

The word stimuli were 160 verbs and 120 nouns. This included four types of verbs: 40 embodied verbs and 40 of each mental, emotional, and nonembodied abstract verbs. The different verb types were differentiated based on ratings obtained for three lexical semantic dimensions (embodiment, cognition, and valence) from Muraki and Pexman (2024). Each verb was in the active, present infinitive form, and had a minimum of 10 valid ratings on each dimension of interest. In addition, all word stimuli (verbs and nouns) were matched (i.e., no significant differences) on lexical semantic dimensions known to influence lexical and semantic processing: word length, word frequency (log word frequency in movie subtitles; Brysbaert and New 2009), and the age of acquisition of the word (Kuperman et al. 2012). The stimuli selection process was completed using the LexOPS package (Taylor et al. 2020) for R (Version 4.2.1; R Core Team 2023). See descriptive statistics in Table S1 for all lexical semantic dimensions and the Appendices 1 and 2 for a full list of word stimuli.

#### 5.5.2 Embodied Verbs

Embodiment ratings were derived from Muraki and Pexman (2024) and based on the definition from Sidhu et al. (2014). They capture the degree to which the meaning of a verb involves the human body, rated on a scale from 1 to 7. The present set of embodied verbs had embodiment ratings greater than 4 (i.e., they were more embodied) whereas all other verb types had embodiment ratings less than 4 (i.e., they were less embodied). The embodied verbs were significantly (*p* < 0.05; see Table S1) higher in embodiment ratings than the other three verb types.

#### 5.5.3 Mental Abstract Verbs

Cognition ratings were derived from Muraki and Pexman (2024) and based on the definition from Muraki et al. (2020). They capture the degree to which the meaning of the verb is related to mental actions or processes of acquiring knowledge and understanding through experience and thought. Words were rated on a scale from 1 to 7. Mental abstract verbs had cognition ratings greater than 5 (i.e., they were more cognitive) and all other verb types had cognition ratings equal to or less than 3.5 (i.e., they were less cognitive). The mental abstract verbs were significantly higher (*p* < 0.05; see Table S1) in cognition ratings than the other three verb types.

#### 5.5.4 Emotional Abstract Verbs

Valence ratings were derived from Muraki and Pexman (2024) and based on the definition from Warriner et al. (2013). They capture the degree to which a verb's meaning is happy versus unhappy. Words were rated on a scale from 1 to 9, where the middle point of the scale (5) indicated that the verb had neutral valence (i.e., neither happy nor unhappy). Emotional abstract verbs had valence ratings less than 4 (i.e., they had negative valence). We selected only negatively valenced verbs, as previous studies have found that positive and negative valence have different behavioral effects on semantic processing (see Ferré et al. 2024 for a recent meta‐analytic review). All other verb types had valence ratings ranging from 4 to 6.5 (i.e., they had neutral valence). The emotional abstract verbs were significantly (*p* < 0.05; see Table S1) lower in valence ratings than were the other three verb types.

#### 5.5.5 Nonembodied Abstract Verbs

The nonembodied abstract verbs were selected to have low embodiment ratings (less than 4), low cognitive ratings (less than or equal to 3.5), and neutral valence ratings (ranging from 4 to 6.5). Therefore, the nonembodied verbs differed significantly (*p* < 0.05; see Table S1) from all the other verb types and specifically on the dimension that defined each of them.

#### 5.5.6 Nouns

Nouns were included to constitute trials requiring a “no” decision in the SJT (i.e., trials where the word was not a verb). Nouns were divided into two types that differed in their association with sensorimotor experience, in order to instill a degree of variability akin to that in the verbs. We used body‐object interaction (BOI) ratings as a semantic dimension analogous to embodiment ratings, because the latter are only available for verbs. BOI (Pexman et al. 2019) describes the ease with which a human body can interact with a word's referent. Half of the nouns in the present study had high BOI ratings (greater than or equal to 4; *n* = 60) and half had low BOI ratings (less than or equal to 3; *n* = 60), and they differed significantly (*p* < 0.05; see Table S1).

#### 5.5.7 Numbers

The number stimuli comprised 64 odd and 64 even numbers (total *N* = 128). The numbers were randomly generated and ranged from five to eight digits in length, with an equal quantity (*n* = 32) of each length in the odd and even number groups.

### 5.6 Experimental Procedure

A PC running PsychoPy (Version 2022.2.4; Peirce et al. 2019) was used for the presentation of stimuli and the recording of responses. Stimuli were presented on a screen positioned outside the bore, which participants viewed using a mirror placed above the head coil. Participants completed four runs, each lasting approximately 7.5 min. Within a run, the SJT and NJT alternated in a block design with a 14 s rest after each number block. There were four SJT blocks containing between 15 and 24 trials (Mean trials = 17.5 per block, block duration range = 56.2 to 99.2 s) while each of the four NJT blocks contained 8 trials (block duration = 20 s). Each trial began with a 500 ms fixation cross (blue cross on number trials, black cross on word trials) presented in the middle of the screen, and then a number or word was presented for 2000 ms in black, lower‐case font on a white background. After the 2000 ms response window, word trials had a variable interstimulus interval (0–13,100 ms; mean ISI = 1840 ms) and number trials had a 0 ms interstimulus interval. Participants were instructed to respond as quickly as possible after the word or number appeared on the screen using two buttons designated as “yes” and “no” on an MR‐compatible response box.

Nested within the block design, we employed a rapid event‐related design for the presentation of the five different word conditions. This was achieved using a pseudorandomized sequence of conditions (1 trial per sequence position) that spanned the four SJT blocks and was used in all four runs. We created four unique lists of 70 words (10 of each verb type and 30 nouns). We matched each word type (embodied, emotional, mental, nonembodied, high BOI nouns and low BOI nouns) on their corresponding semantic variable across lists (e.g., the four lists did not significantly differ on the valence ratings of the emotional abstract verbs within the list). We also matched across lists for the lexical variables length, frequency, and age of acquisition. Each list was used once across the four runs. The order in which lists of words were presented was counterbalanced across participants using a balanced Latin square design. The exact word for each trial was randomly selected from a condition‐specific sub‐list, such that no pair of participants would have seen the words in the same order. In a similar fashion, four unique lists of 32 numbers were created, and the order in which they were presented was counterbalanced across participants. All study materials are available for download on our OSF project page https://osf.io/vd9ba/.

### 5.7 Imaging Acquisition

All imaging was performed on a 3.0 T Phillips Elition MRI scanner with a 32‐channel head coil and a SENSE factor of 2.5. We used a dual‐echo gradient‐echo EPI fMRI sequence to acquire 31 axial slices covering the whole brain in ascending sequential order with the following parameters: short echo time (TE) = 12 ms and long TE = 35 ms, repetition time (TR) = 2000 ms, and flip angle = 85°. The functional EPIs were acquired with an in‐plane reconstructed resolution of 2.5 × 2.5 mm and a slice thickness of 4 mm voxels (reconstruction matrix = 96 × 96; FOV (mm) = 240 × 240 × 124). We acquired five dummy scans prior to each run (to develop a steady state magnetisation), followed by 224 volumes per run. We acquired the four main functional runs with a single direction k‐space traversal in the left–right phase‐encoding direction. In addition, we acquired a short EPI “pre‐scan” with the participants at rest. The parameters of the pre‐scan matched the main functional scans with the exception that they included interleaved dual direction k‐space traversals. This provided us with 10 pairs of images with opposing direction distortions (10 left–right and 10 right–left) which were used in a k‐space distortion correction procedure for addressing geometric distortion and mislocalisation due to magnetic susceptibility artifacts. We acquired a high‐resolution T2‐weighted scan to assess the accuracy of our distortion correction procedure, which contained 36 slices covering the whole brain with the following parameters: TE = 78 ms, TR = 3410 ms; flip angle = 90°. The T2‐weighted scan was acquired with a reconstructed voxel size (mm) of 0.6 × 0.6 × 4 and a slice thickness of 4 mm voxels with a 1 mm slice gap (reconstruction matrix = 400 × 370; FOV (mm) = 240 × 240 × 179). In addition, we used a T1‐weighted imaging sequence to acquire an anatomical image used to estimate the transformation of our functional images into MNI space. The T1 images contained 175 slices covering the whole brain and were acquired with the following parameters: TE = 18 ms, TR = 3.5 ms; flip angle = 8°, reconstructed voxel size (mm) = 0.94 × 0.94 × 1 and reconstruction matrix = 240 × 240. All images were acquired with a 30° axial tilt.

### 5.8 Data Analysis

#### 5.8.1 Data Cleaning

Two participants had number judgment task accuracy values which indicated that they had reversed the buttons used for their response (e.g., accuracy less than 10%), so their number judgment task data were recoded.

#### 5.8.2 Behavioral Data

Behavioral analyses were conducted using the statistical software package R (R Core Team 2023). Task accuracy and response time were assessed using linear mixed effect models which included random effects of participant and item and fixed effects of word or trial type. We excluded practice trials, trials where the response time was more than three SD away from the participant's mean response time, and words with significantly lower than chance accuracy on the SCT. We only included correct trials in our response time models. Inferential statistics were calculated to compare performance in the SCT and NJT, as well as to compare between verb types in the SCT and odd and even numbers in the NJT.

#### 5.8.3 fMRI Distortion Correction and Preprocessing

fMRI preprocessing and analyses were conducted using the statistical software package MATLAB 2019b (for distortion correction; The MathWorks Inc. 2019) and 2020a (The MathWorks Inc. 2020), SPM12, and the CoSMo MVPA toolbox for MATLAB (Oosterhof et al. 2016). A spatial remapping correction was computed separately for images acquired at the long and the short echo time using the method reported in Embleton et al. (2010), implemented via an in‐house MATLAB script (available upon request), as well as SPM12's (Statistical Parametric Mapping software; Wellcome Trust Centre for Neuroimaging, London, UK) 6‐parameter rigid body registration algorithm. In the first step, each functional volume was registered to the mean of the 10 pre‐scan volumes acquired at the same echo time. This step is both a required part of the distortion correction procedure and corrects the timeseries for differences in participant positioning in between functional runs and for minor motion artefacts within a run. Next, one spatial transformation matrix per echo time was calculated from the opposingly distorted pre‐scan images. These transformations consisted of the remapping needed to correct geometric distortion and were then applied to each of the main functional volumes. This resulted in two motion and distortion‐corrected time‐series per run (224 volumes per echo per run), which were subsequently combined at each timepoint using a simple linear average of image pairs.

The following preprocessing steps were completed using SPM12 in Matlab 2020a. Slice‐timing correction was performed, referenced to the middle slice. The T1‐weighted anatomical image was co‐registered to a mean of the distortion‐ and motion‐corrected images using a six‐parameter rigid‐body transform and the normalized mutual information objective function. SPM12's unified segmentation and normalization function was used to estimate a spatial transform to register the anatomical image to Montreal Neurological Institute (MNI) standard stereotaxic space and this transform was subsequently applied to the co‐registered functional volumes which were then resampled to a 2 × 2 × 2 mm voxel size. As a final step, the normalized functional images were smoothed using an 8 mm full‐width half‐maximum Gaussian filter.

#### 5.8.4 Univariate Analyses

Functional data were analyzed using the general linear model approach (GLM). We conducted within‐subjects fixed effects analyses with all functional runs incorporated into a single GLM per participant. Onsets for each of the verb types, nouns, and number blocks were modeled and convolved with the canonical hemodynamic response function, and a high‐pass filter (cutoff of 128 s) was applied. The extracted motion parameters were also entered into the GLM model as regressors of no interest. We restricted the analyses to grey matter using an explicit mask generated from a group‐level probabilistic tissue segment arising out of the SPM12 unified segmentation and normalization procedure, which was binarized with a 0.4 threshold.

We performed pre‐registered confirmatory analyses that tested two hypotheses: (1) that different verb types will differentially recruit a distributed set of cortical regions outside the ATL consistent with multiple representation theories of semantic representation, and (2) that all verb types will recruit the vATL consistent with the hub‐and‐spoke model of semantic memory. We conducted whole brain analyses and selected a priori regions of interest (ROI) based on other studies that have examined the neural correlates of abstract word meaning. At the group‐level analysis, we examined whole brain contrasts between each verb type relative to the active (NJT) baseline, as well as each of the abstract verb types relative to the embodied verbs, to examine whether there were regions specific to processing abstract verb meaning above and beyond processing verb meaning generally. Whole brain analyses of each verb type relative to embodied verbs also served as an exploratory test of whether ATL subregions respond differentially to these verb types. While the graded hub hypothesis would predict such differences, we did not have clear hypotheses regarding where in the ATL they would occur. It is also possible that differences do not manifest in terms of magnitude of engagement, but rather in patterns of activation. For this reason we used an “information‐based” classifier analysis to complement the univariate approach to testing this hypothesis (see below).

Whole‐brain multi‐subject random effects analyses were conducted on each of the following contrasts of interest: embodied verbs > numbers, mental verbs > numbers, emotional verbs > numbers, nonembodied verbs > numbers, mental verbs > embodied verbs, emotional verbs > embodied verbs, and nonembodied verbs > embodied verbs. We performed one‐sample *t*‐tests on all these sets of contrast images, restricting the statistical maps to cerebral tissue using the same explicit group‐level mask as used in the single subject analyses. Cluster‐wise statistical significance was assessed using a cluster‐defining voxel height threshold of *p* < 0.001 uncorrected, and a family‐wise error (FWE) corrected cluster extent threshold at *p* < 0.05. The resulting thresholded maps were overlaid on a MNI152 template brain in MRIcroGL for all figures (Rorden and Brett 2000). We used the label4MRI package implemented in R (Chuang 2024) to inform the labelling of each cluster based on its peak coordinates.

To increase sensitivity to the hypothesized effects and complement the whole‐brain analyses, we examined a priori ROI to extract and quantify the magnitude of activation within different cortical and ATL subregions. The ROI analysis was implemented using the SPM MarsBar toolbox (Brett et al. 2002). We defined seven ROIs based on coordinates extracted from published meta‐analyses or fMRI studies of semantic processing. Coordinates were used to define the center of mass of spherical ROIs with radii of 8 mm (Volume = 2056 mm^3^). One of these ROIs corresponds to the center‐point of the graded ATL semantic hub, located in the ventral ATL, and was based on the findings of Binney et al. (2010). The other six spherical ROIs correspond to brain regions that might, based on prior findings, be differentially recruited by semantic processing of different verb types, as detailed in the following paragraph (also see Table 1).

**TABLE 1:** Spherical regions of interest (ROI) by contrast.

ROI location	Contrast	Centre of mass (MNI)		
		*x*	*y*	*z*
Left anterior supramarginal gyrus	Embodied > nonembodied	−60	−30	30
Left posterior superior temporal sulcus	Embodied > nonembodied	−62	−56	8
Left amygdala	Emotional > embodied	−26	−6	−22
Right amygdala	Emotional > embodied	24	−4	−28
Left temporal parietal junction	Mental > embodied	−50	−58	20
Left middle temporal gyrus	Nonembodied > embodied	−61	−44	−2
Ventral anterior temporal lobe	Embodied > number Emotional > number Mental > number Nonembodied > number	−36	−15	−30

For the embodied verbs, we selected regions associated with conceptual processing related to action and motion from the Kuhnke et al. (2023) meta‐analysis that examined modality‐specific brain regions that were uniquely activated by processing particular types of concepts. We selected ROIs with the highest specificity for action and motion concepts in their contrast analyses assessing modality specificity. For action concepts, this was a region in the left anterior supramarginal gyrus, and for motion, this was an area in the left posterior superior temporal sulcus. For the emotional verbs, we selected regions associated with conceptual processing related to emotion from the Kuhnke et al. (2023) meta‐analysis. We selected ROIs with the highest specificity for emotional concepts in their contrast analyses assessing modality specificity. This was located outside the anterior temporal cortex, in the bilateral amygdalae. For the mental verbs, we selected a region of the temporal parietal junction associated with processing theory‐of‐mind‐related concepts by the Desai et al. (2018) meta‐analysis. We anticipated that the nonembodied verbs would be the most abstract, as they have no relation to physical or interoceptive experiences of the body, so we selected a region associated with processing abstract concepts in the Bucur and Papagno (2021) meta‐analysis. The middle temporal gyrus has also been associated with language comprehension in previous research (see Dronkers et al. 2004), which is consistent with the proposal that abstract concepts that lack sensory or motor experiences may rely more on language to ground their meaning.

#### 5.8.5 Multivariate Analyses

Finally, we used a constrained searchlight multivoxel pattern analysis (MVPA) to explore whether patterns of activity across the ATL could be used to classify the different verb types. The analysis used a take‐one‐run‐out cross‐validation and a linear discriminant analysis (LDA) classifier implemented via the CoSMo MVPA toolbox (Oosterhof et al. 2016). For each participant, the same GLM as used for univariate analyses was estimated, but this time on unsmoothed image data, and *β* weights were extracted for each verb condition. The *β* weights were then submitted to a searchlight method MVPA restricted to the ATL, using a mask previously described by Hung et al. (2020). This mask defined a volume of interest that covered the anterior portion of the temporal lobe, including the temporal pole, anterior superior temporal gyrus, anterior middle temporal gyrus, anterior inferior temporal gyrus, anterior temporal fusiform cortex, and anterior parahippocampal gyrus. The posterior boundary of the ATL was approximately MNI coordinate *y* = −24 along the ventral surface and *y* = −14 on the lateral surface, and the volume was 9706 voxels (voxel size = 2 × 2 × 2 mm^3^). The searchlight was defined with a spherical neighborhood comprising the 100 voxels nearest to the centre voxel. We assessed z‐transformed classification accuracy results between mental versus embodied verbs, emotional versus embodied verbs, and nonembodied versus embodied verbs. Cluster‐wise statistical significance was assessed using a cluster‐defining voxel height threshold of *p* < 0.005 uncorrected and a FWE corrected cluster extent threshold at *p* < 0.05. We selected a less stringent cluster‐defining threshold in the MVPA analysis to improve sensitivity given the limited set of stimuli in each condition.

## 6 Results

### 6.1 Behavioral Analyses

The descriptive statistics for both the SCT and the NJT are presented in Table 2. For two verbs (*introspect*, *impoverish*) response accuracy was significantly below chance, and so these verbs were removed from the analyses. In the SCT, there were no significant effects of verb type on response time or accuracy (as referenced against embodied verbs; see Table S2 for both models). To follow up, we performed pairwise comparisons with a Tukey correction. There were no significant differences in response time for any pairwise comparison. There was one significant difference in accuracy: Emotional abstract verbs were responded to less accurately than mental abstract verbs (*z* = −2.92, adjusted *p* = 0.019). Responses to the SCT were significantly slower and less accurate than the NJT (see Table S3 for both models).

**TABLE 2:** Response time and accuracy descriptive statistics by task and trial type.

Task and trial type	*M* participant RT in milliseconds (SD)	*M* participant accuracy in % (SD)
Syntactic classification task		
All trials	924.40 (133.49)	90.48 (8.44)
Embodied verbs	906.44 (158.21)	90.63 (12.69)
Emotional verbs	920.86 (148.85)	87.82 (14.63)
Mental verbs	898.02 (160.66)	92.12 (13.02)
Nonembodied verbs	920.66 (156.63)	88.93 (14.20)
Nouns	948.24 (125.47)	91.28 (7.33)
Number judgment task		
All trials	830.90 (198.09)	93.22 (8.02)
Even	841.03 (208.90)	89.90 (14.04)
Odd	820.77 (192.00)	96.54 (3.79)

### 6.2 Univariate fMRI Analyses

#### 6.2.1 Whole Brain Contrasts

**FIGURE 1:**
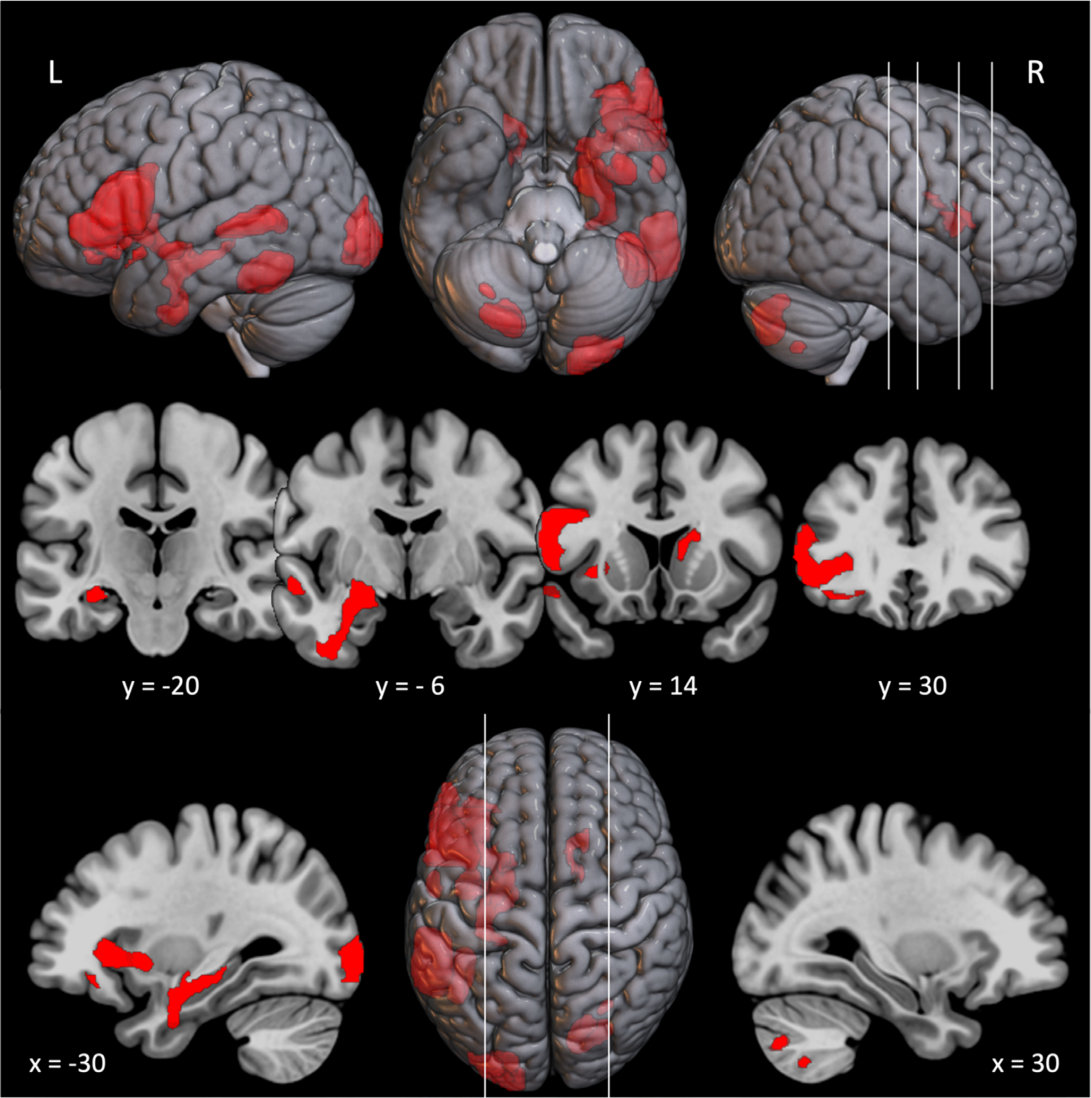
Cortical regions activated by syntactic classification > number judgment. Cortical regions activated during the syntactic classification task relative to the number judgment task. The statistical maps were independently thresholded with an uncorrected voxel height threshold of *p* < 0.001 and a FWE‐corrected minimum extent cluster threshold *p* < 0.05. Cross sections were chosen to display activation in the semantic network based on the large cluster reported in Table 3, extending up from the ventral ATL [−30, −20, −12] into the IFG [−46, 30, 2]. White lines represent locations of coronal and sagittal slices.

Whole brain univariate analyses were conducted to contrast (i) the SCT with the active baseline (number judgment), (ii) each verb type with the active baseline, and (iii) each abstract verb type with embodied verbs. Compared to the baseline task, the SCT was associated with robust activation in the semantic network (see Figure 1 and Table 3). This included a large cluster that extended up from the vATL (*x* ≈ −30) into the left ventrolateral prefrontal cortex encompassing regions of the pars orbitalis and the pars triangularis. There were also clusters of activation extending along the anterior superior temporal gyrus, posterior middle, and inferior temporal gyri. In addition, we observed activation in the left occipital lobe, possibly due to the greater visual complexity of word compared to number stimuli (Binney et al. 2016) as well as activation in the right cerebellum.

**TABLE 3:** significant activation clusters for syntactic classification relative to number judgment.

Cluster name and location of maximum	Cluster extent (voxels)	Peak (*Z*)	Maxima MNI coordinates (mm)		
			*x*	*y*	*z*
**R cerebellum**					
Lobule VIIB	**699**	6.88	24	−76	−46
Crus I		5.19	20	−72	−30
Crus II		4.90	14	−80	−36
Lobule VIII		4.36	28	−64	−52
**L fronto‐temporal**					
Pars opercularis	**4021**	6.16	−54	14	20
Pars triangularis		5.87	−46	30	2
Insula		5.63	−34	24	0
Hippocampus		5.38	−20	−8	−12
Pars triangularis		5.34	−50	42	4
Pars orbitalis		5.2	−36	34	−10
Anterior ITG		5.16	−42	−6	−38
Hippocampus		4.8	−30	−20	−12
**L occipital lobe**					
Inferior occipital gyrus	**975**	5.51	−22	−90	−10
Middle occipital gyrus		4.91	−28	−98	−6
Inferior occipital gyrus		4.80	−20	−102	−10
Middle occipital gyrus		3.68	−12	−96	2
**L posterior temporal lobe**					
Posterior ITG	**773**	5.43	−42	−48	−16
Cerebellum		4.21	−38	−40	−26
Fusiform gyrus		3.43	−40	−68	−14
**L posterolateral temporal lobe**					
Posterior STS/MTG	**880**	5.12	−48	−40	4
Posterior MTG		4.89	−60	−42	4
**R basal ganglia**					
Caudate nucleus	**289**	4.77	12	16	10
Caudate nucleus		4.39	20	16	14
Caudate nucleus		4.18	12	2	18
Caudate nucleus		4.03	10	6	4
Caudate nucleus		3.88	20	−14	24
**L anterior temporal lobe**					
Anterior STG	**297**	4.57	−54	−6	−10
Temporal pole		3.93	−54	14	−10
Temporal pole		3.71	−50	18	−16

*Note:* Significant activation clusters in the syntactic classification minus number judgment contrast identified at *p* < 0.05, FWE‐corrected, resulting in an extent threshold of *k* = 289 voxels, after a cluster‐defining threshold of *p* < 0.001 uncorrected. This table shows up to 8 local maxima more than 8 mm apart in each cluster.

**FIGURE 2:**
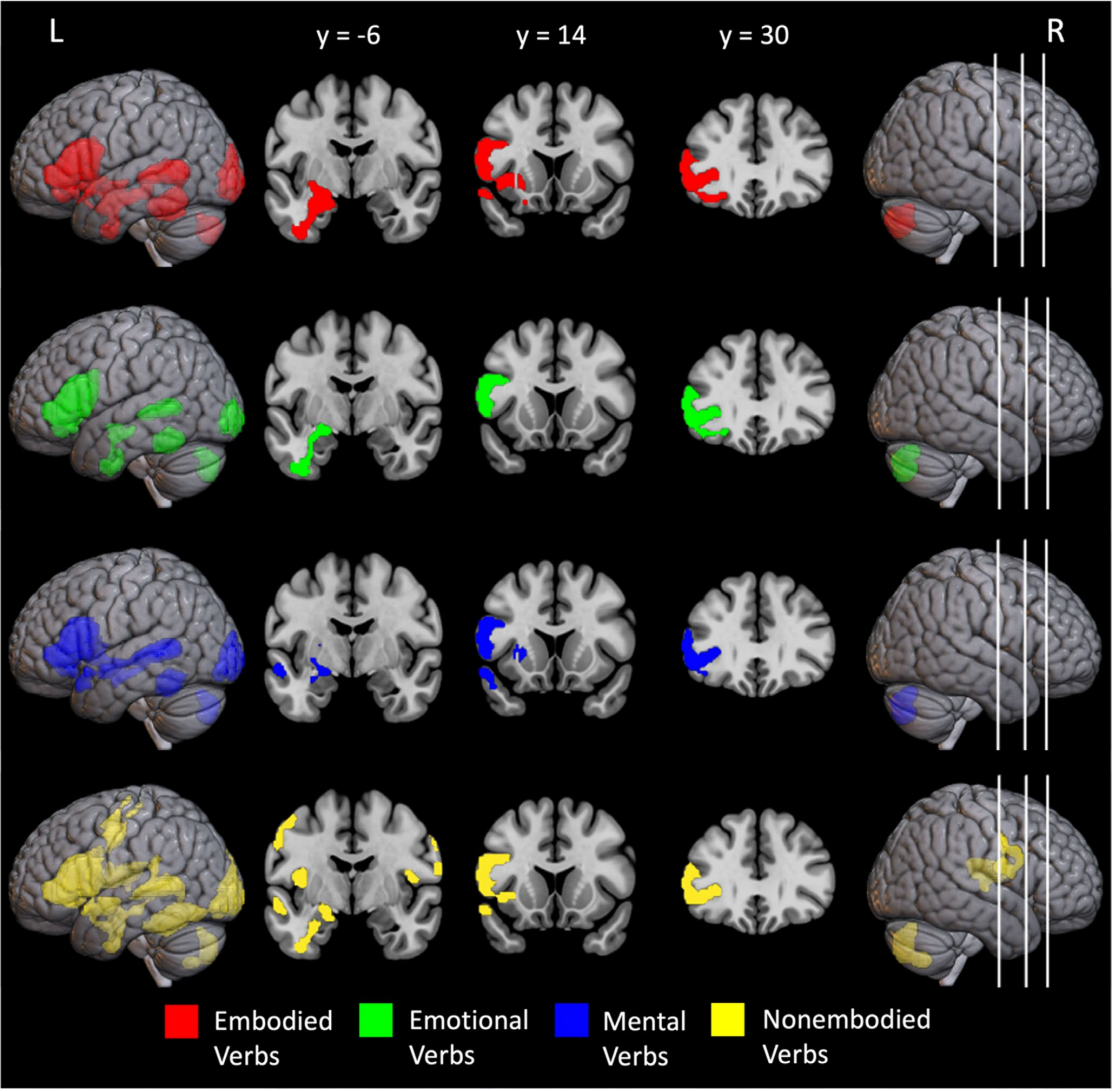
Cortical regions activated by each verb type > number judgment. Cortical regions activated during the verb trials of the syntactic classification task relative to the number judgment task. The statistical maps were independently thresholded with an uncorrected voxel height threshold of *p* < 0.001 and a FWE‐corrected minimum extent cluster threshold *p* < 0.05. Regions activated to only embodied verbs are presented in red, to only emotional verbs in green, to mental verbs in blue, and to nonembodied verbs in yellow. Cross sections were chosen to display activation in the semantic network based on the frontal and anterior temporal cluster reported in Table 4, extending up from the ventral ATL [−18, −6, −12] into the IFG [−56, 14, 20] and [−42, 30, 2]. White lines represent locations of coronal slices.

The contrasts comparing each verb type to the baseline task show a similar pattern of activation to the contrast comparing all words with the baseline task, with a few exceptions (see Figure 2 and Table 4). All verbs were associated with activity in the inferior frontal gyrus (IFG) and posterior middle temporal gyrus (pMTG), both core regions in the semantic network. Emotional, embodied, and nonembodied verbs were associated with activity in the vATL, yet mental verbs were not. Embodied verbs were associated with a larger cluster of activation in the left IFG and with activation extending more posterior on the MTG than any other verb condition. Similarly, nonembodied verbs were associated with larger clusters of activation in the left IFG and the posterior portion of the left MTG and fusiform gyri. They were also uniquely associated with activity in the left motor cortex and the left and right somatosensory cortices. There were no significant differences in activation between each abstract verb type compared to the embodied verbs after corrections, suggesting that they showed a similar pattern of activation. However, there was one cluster in the left motor cortex that was more active for nonembodied compared to embodied verbs that approached significance (*p* = 0.069 FWE‐corrected, resulting in an extent threshold of *k* = 133 voxels).

**TABLE 4:** Significant activation clusters in the whole brain analyses.

Contrast	Cluster name and location of maximum	Cluster extent (voxels)	Peak (*Z*)	Maxima MNI coordinates (mm)		
				*x*	*y*	*z*
Embodied verbs > number judgment	**R cerebellum**					
	Crus II	**677**	6.21	24	−76	−44
	**L fronto‐temporal**					
	Pars opercularis	**4290**	6.05	−56	14	20
	Pars triangularis		5.96	−42	30	2
	Hippocampus		5.40	−18	−6	−12
	Pars triangularis		5.35	−50	42	2
	Hippocampus		5.33	−28	−18	−12
	Pars triangularis		5.24	−34	34	−10
	Parahippocampal gyrus		5.19	−30	−26	−10
	Hippocampus		4.94	−26	−4	−22
	Putamen		4.79	−28	12	−2
	Anterior ITG		4.69	−38	−4	−36
	**L posterior temporal lobe**					
	Fusiform gyrus	**1968**	5.60	−44	−48	−18
	MTG		5.09	−50	−44	2
	MTG		5.01	−50	−58	2
	**L occipital lobe**					
	Inferior occipital gyrus	**931**	5.21	−20	−90	−10
	Middle occipital gyrus		4.65	−22	−94	−2
	Middle occipital gyrus		4.55	−24	−96	14
	Inferior occipital gyrus		4.39	−28	−90	−6
	Middle occipital gyrus		3.91	−12	−96	2
Emotional verbs > number judgment	**R cerebellum**					
	Crus II	**543**	6.19	22	−78	−46
	Crus II		4.81	14	−80	−34
	Crus I		4.80	18	−72	−30
	**L inferior frontal lobe**					
	Pars opercularis	**2242**	6.05	−50	14	24
	Pars triangularis		5.96	−48	26	0
	Pars triangularis		5.91	−52	22	20
	Pars orbitalis		5.07	−38	34	−10
	Pars orbitalis		3.80	−26	30	−12
	Pars orbitalis		3.24	−20	22	−18
	**L anterior temporal lobe**					
	Anterior ITG	**621**	5.39	−42	−8	−38
	Hippocampus		4.59	−24	−12	−12

	**L posterior temporal lobe**					
	Fusiform gyrus	**419**	5.00	−42	−46	−18
	**L occipital lobe**					
	Inferior occipital gyrus	**662**	4.98	−20	−92	−10
	Middle occipital gyrus		4.81	−28	−98	−4
	Lingual gyrus		4.36	−18	−102	−12
	**L posterolateral temporal lobe**					
	STS	**638**	5.91	−48	−38	4
STS/MTG	5.26		−56	−48	4
Mental verbs > number judgment	**R cerebellum**					
	Lobule VIIB	**438**	6.12	24	−76	−46
	Crus I		4.20	18	−74	−28
	**L fronto‐temporal**					
	Pars opercularis	**2614**	5.89	−54	14	20
	Pars triangularis		5.30	−48	24	0
	Insula		4.91	−34	26	6
	Pars triangularis		4.65	−52	38	2
	Putamen		4.33	−26	14	2
	Hippocampus		4.2	−20	−6	−10
	Hippocampus		4.2	−30	−20	−12
	Pars triangularis		4.01	−38	34	−10
	**L occipital lobe**					
	Inferior occipital gyrus	**958**	5.46	−22	−100	−12
	Middle occipital gyrus		5.38	−22	−98	−2
	Middle occipital gyrus		3.60	−12	−96	2
	**L temporal lobe**					
	Posterior MTG	**1256**	5.45	−50	−40	2
	Posterior MTG		5.26	−60	−42	4
	Anterior STG		4.26	−54	−6	−10
	Temporal pole		4.11	−48	18	−16
	Anterior STS/MTG		3.86	−54	−16	−6
	**L posterior temporal lobe**					
	Fusiform gyrus	**393**	6.18	−40	−48	−16
Nonembodied verbs > number judgment	**R cerebellum**					
	Lobule VIIB	**530**	6.22	20	−78	−46
	Lobule VIII		4.62	28	−62	−52
	Crus I		3.79	20	−72	−28

	**L fronto‐temporal**					
	Pars opercularis	**3377**	5.97	−52	12	20
	Pars triangularis		5.67	−50	24	18
	Pars triangularis		5.61	−50	26	2
	Pars triangularis		5.46	−50	34	4
	Insula		5.27	−34	24	0
	Anterior fusiform gyrus		4.68	−38	−8	−36
	Hippocampus		4.36	−18	−12	−16
	Anterior STG/STS		4.36	−54	−6	−10
	Amygdala		3.93	−26	0	−26
	Temporal pole		3.85	−52	10	−12
	**L temporal‐occipital**					
	Fusiform gyrus	**3782**	5.60	−42	−48	−18
	Inferior occipital gyrus		5.43	−22	−90	−10
	MTG		5.00	−50	−40	2
	STS/MTG		4.84	−60	−40	4
	Insula		4.47	−36	−20	18
	MTG		4.39	−48	−58	0
	Cerebellum		4.31	−38	−38	−28
	Fusiform gyrus		4.24	−40	−68	−14
	Inferior occipital gyrus		4.06	−36	−86	−6
	Middle occipital gyrus		3.92	−12	−96	2
	**L fronto‐parietal**					
	Precentral gyrus	**398**	5.09	−42	−12	58
	Postcentral gyrus		4.91	−52	−12	48
	Precentral gyrus		4.74	−38	−18	66
	Precentral gyrus		4.02	−40	−26	66
	**R fronto‐parietal–temporal**					
	Rolandic operculum	**565**	4.00	44	−12	18
	Postcentral gyrus		3.70	62	−4	38
	STG		3.67	44	−18	4
	Insula		3.64	40	−24	14
	Precentral gyrus		3.57	62	6	28
	Rolandic operculum		3.55	38	−18	22
	Postcentral gyrus		3.48	66	−6	18
Rolandic operculum	3.26		62	2	12

*Note:* Significant activation clusters in the embodied verbs minus number judgment contrast identified at *p* < 0.05, FWE‐corrected, resulting in an extent threshold of *k* = 677 voxels, after a cluster‐defining threshold of *p* < 0.001 uncorrected. Significant activation clusters in the emotional verbs minus number judgment contrast identified at *p* < 0.05, FWE‐corrected, resulting in an extent threshold of *k* = 419 voxels, after a cluster‐defining threshold of *p* < 0.001 uncorrected. Significant activation clusters in the mental verbs minus number judgment contrast identified at *p* < 0.05, FWE‐corrected, resulting in an extent threshold of *k* = 393 voxels, after a cluster‐defining threshold of *p* < 0.001 uncorrected. Significant clusters in the nonembodied verbs minus number judgment contrast identified at *p* < 0.05, FWE‐corrected, resulting in an extent threshold of *k* = 398 voxels, after a cluster‐defining threshold of *p* < 0.001 uncorrected. Table shows up to 10 local maxima more than 8 mm apart in each cluster.

#### 6.2.2 ROI Analyses

**FIGURE 3:**
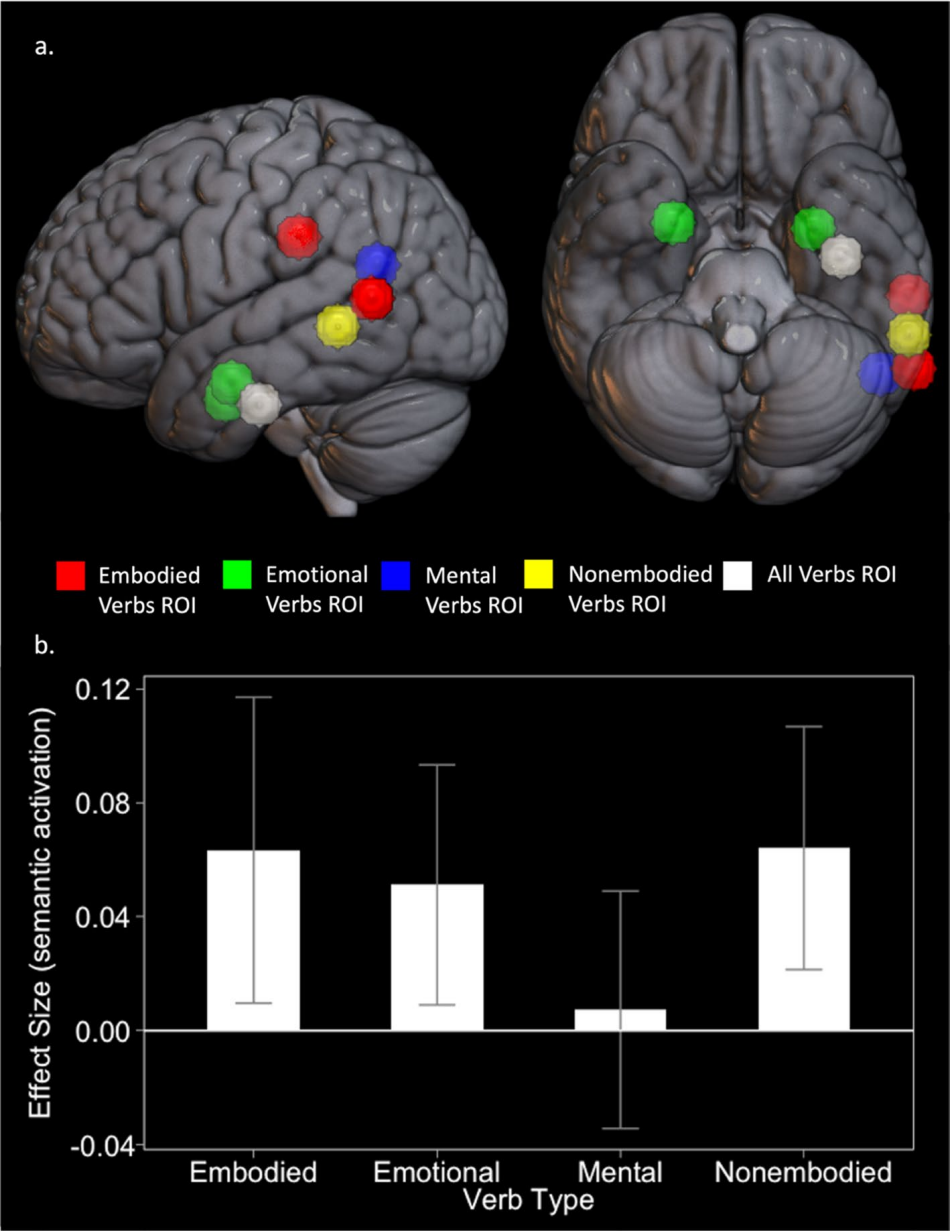
(a) Spherical region of interest locations. (b) Effect size estimates for verb > number contrasts in the vATL. vATL, ventral anterior temporal lobe. (a) A priori spherical regions of interest (ROI). Red shows the left anterior supramarginal gyrus ROI and the left posterior superior temporal sulcus ROI (both previously associated with processing action and motion concepts; Kuhnke et al. 2023), green shows the left and right amygdala ROIs (both previously associated with processing emotional concepts; Kuhnke et al. 2023), blue shows the left temporal parietal junction ROI (previously associated with processing theory of mind concepts; Desai et al. 2018), yellow shows the left middle temporal gyrus ROI (previously associated with processing abstract concepts; Bucur and Papagno 2021), and white shows the left ventral anterior temporal lobe ROI (previously identified as the centre‐point of the ATL semantic hub; Binney et al. 2010). (b) Results for the ventral ATL ROI analysis. Bars show relative activation for each verb type compared to the number judgment task. Error bars show standard error.

Next, we conducted a series of focused a priori ROI analyses (see Figure 3a for locations and descriptions). The ROIs were defined based on peak activation coordinates from previous literature and were selected to correspond to the semantic categories of our verb stimuli, as well as semantic processing more generally. In the vATL, we observed significant activation for all verb types in contrast to the number judgment task, apart from the mental verbs (Figure 3b) that did not activate the vATL to a significantly greater degree than in the number judgment task. Engagement of the vATL by mental state verbs (relative to the NJT) was not significantly different from that evoked by embodied verbs. This suggests that vATL activation was simply not as robust for mental verbs as it was for other verbs (compared to baseline). We observed no significant differences in activation when comparing abstract verb types to embodied verbs in ROIs previously associated with emotional, mental, and nonembodied information (Table 5). When comparing the embodied verbs to nonembodied verbs, activity in the left posterior STS was marginally significant (*p* = 0.051). This ROI has been associated with processing motion‐related concepts (Kuhnke et al. 2023).

**TABLE 5:** Region of interest contrast results.

Contrast	ROI	*t*	*p*
Embodied > nonembodied	L anterior supramarginal gyrus	1.00	0.163
	L posterior superior temporal sulcus	1.70	0.051
Emotional > embodied	L amygdala	−3.00	0.997
	R amygdala	−2.85	0.996
Mental > embodied	L temporal parietal junction	−1.76	0.955
Nonembodied > embodied	L middle temporal gyrus	−0.90	0.813
Embodied > number	L ventral ATL	1.94	0.031*
Emotional > number	L ventral ATL	2.00	0.028*
Mental > number	L ventral ATL	0.29	0.387
Nonembodied > number	L ventral ATL	2.47	0.010*
Mental > embodied	L ventral ATL	−2.65	0.993
Emotional > embodied	L ventral ATL	−0.65	0.739
Nonembodied > embodied	L ventral ATL	0.03	0.489

Note:* indicates *p* < 0.05.

Abbreviations: ATL, anterior temporal lobe; L, left; R, right.

#### 6.2.3 Multivariate Analyses

**FIGURE 4:**
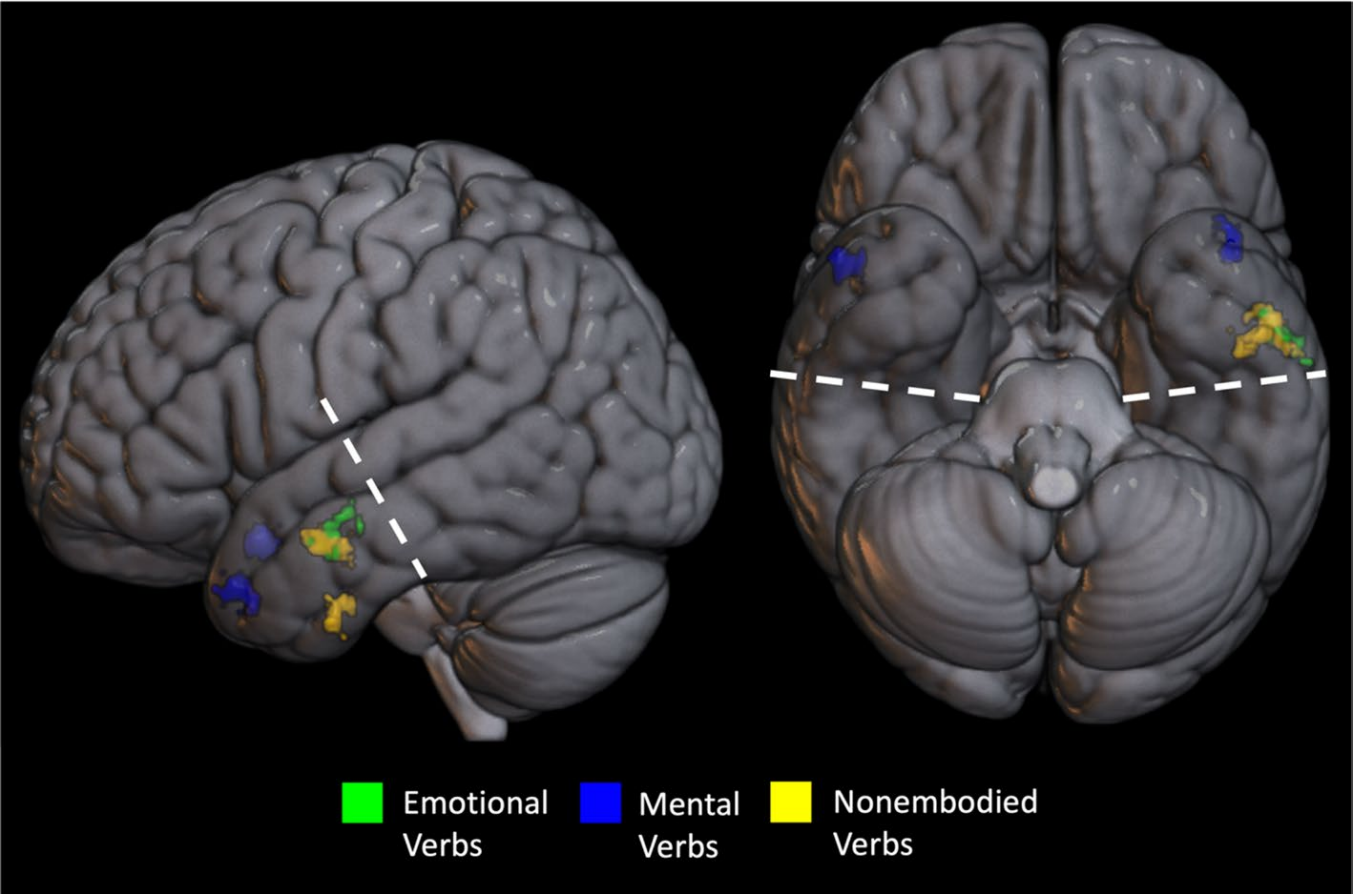
Cortical regions associated with significantly higher classification accuracies in the MVPA. Green indicates regions involved in classifying emotional relative to embodied verbs. Blue indicates regions involved in classifying mental relative to embodied verbs. Yellow indicates regions involved in classifying nonembodied relative to embodied verbs. The statistical map was thresholded with an uncorrected voxel height threshold of *p* < 0.005 and a FWE‐corrected minimum extent cluster threshold at *p* < 0.05 for each contrast. Dotted lines represent the approximate posterior boundary of the ATL volume to which the MVPA was restricted (approximately MNI coordinate *y* = −24 along the ventral surface and *y* = −14 on the lateral surface).

A searchlight MVPA restricted to the ATL revealed clusters sensitive to differences between each abstract verb type and embodied verbs (Figure 4 and Table 6). A group of voxels in the bilateral dorsolateral temporal poles was associated with high classification accuracy when differentiating between mental verbs and embodied verbs. A group of voxels in the left middle temporal gyrus in the posterior portion of the ATL was associated with high classification accuracy when differentiating between emotional and embodied verbs. A similarly located group of voxels in the left middle and inferior temporal gyri in the posterior portion of the ATL was associated with high classification accuracy when differentiating between nonembodied and embodied verbs.

**TABLE 6:** Significant clusters associated with classification accuracy in MVPA.

Classifier	Cluster name and location of maximum	Cluster extent (voxels)	Peak (*Z*)	Maxima MNI coordinates (mm)		
				*x*	*y*	*z*
Emotional versus embodied verbs	L MTG	**36**	3.57	−54	−4	−20
			3.21	−62	−8	−14
Mental versus embodied verbs	R temporal pole	**35**	3.81	54	10	−20
	L temporal pole	**31**	3.54	−44	12	−34
			3.31	−42	22	−32
Nonembodied versus embodied verbs	L MTG/ITG	**40**	4.03	−54	−4	−22
			2.96	−60	−12	−26
	L ITG	**32**	3.57	−46	−12	−36
			3.07	−52	−6	−42

*Note:* Significant clusters in emotional versus embodied verb classification accuracy identified at *p* < 0.05, FWE‐corrected, resulting in an extent threshold of *k* = 36 voxels, after a cluster‐defining threshold of *p* < 0.005 uncorrected. Significant clusters in mental versus embodied verb classification accuracy identified at *p* < 0.05, FWE‐corrected, resulting in an extent threshold of *k* = 31 voxels, after a cluster‐defining threshold of *p* < 0.005 uncorrected. Significant n clusters in nonembodied versus embodied verb classification accuracy identified at *p* < 0.05, FWE‐corrected, resulting in an extent threshold of *k* = 32 voxels, after a cluster‐defining threshold of *p* < 0.005 uncorrected. Table shows up to 2 local maxima more than 8 mm apart in each cluster.

## 7 Discussion

The potential of the hub‐and‐spoke hypothesis to account for verb processing, and particularly abstract verb processing, has not previously been investigated with fMRI. This was the aim of the present study. The key findings were as follows.

1. The vATL was engaged in semantic processing of all verb types (although to a lesser extent for mental state verbs), confirming its central role in the representation of concepts of all types. This is the first demonstration using distortion‐corrected fMRI of vATL involvement in verb processing.
2. Outside of the ATL, we observed robust activity within other parts of the core semantic cognition network, including the left IFG and the posterior MTG, for all verb types.
3. We found that ATL subregions other than the vATL were differentially associated with processing the three types of abstract verbs.
4. We observed only modest differences between verb types in cortical activity outside of the core semantic network. Contrary to the hypotheses, nonembodied abstract verbs (e.g., *reduce*) were associated with activity in the motor and somatosensory cortices. Embodied verbs (e.g., *straddle*) were associated with activity in the MTG that extended more posteriorly than other verb types, and with the posterior STG, at least to a greater extent than nonembodied verbs (though these differences did not reach *p* < 0.05).

These findings support the proposal that the vATL is a supramodal hub for semantic representations and provide new insight into the role of the wider ATL region in abstract verb processing. We found limited evidence for differential activity associated with different verb types across distributed “spoke” regions and some observed differences were contradictory to the hypotheses (which we consider in greater detail below). However, differential activity observed within the ATL suggests that the kinds of information converging within the ATL are dependent on verb type, and this manifests in graded patterns that are consistent with the graded hub hypothesis.

### 7.1 The vATL Supramodal Semantic Hub and Semantic Cognition Network

The whole brain analyses revealed activity in the vATL during the language task when contrasted to the baseline number judgment task, as well as in each of the contrasts between the embodied, emotional, and nonembodied verbs and the baseline task. This confirms a role of the vATL in processing concepts of all types, and not just nouns or concrete concepts (Yi et al. 2007). Previous research employing a range of neuroscience methods has converged on the vATL as a core supramodal semantic hub (Bajada et al. 2019; Binney et al. 2016; Hoffman et al. 2015; Rice et al. 2018) and atrophy of the vATL is associated with profound semantic impairment (Patterson et al. 2007). However, most of this research has focused on concrete nouns, and the role of the vATL in representing abstract concepts and verbs has been less clear.

The neuropsychological data present a conflicted picture, as illustrated by a recent scoping review of abstract versus concrete word processing deficits in patients with vATL atrophy (i.e., semantic dementia); many studies have found that abstract word processing is more preserved than concrete word processing, which suggests the vATL is less important for the representation of abstract concepts, while some studies found the opposite (more preserved concrete word processing) and others found no difference (Mancano and Papagno 2023). These inconsistencies may reflect varying degrees of control for word frequency, individual differences between patients in word familiarity, as well as idiosyncrasies in the distributions of atrophy present in some case studies (Hoffman 2016). Recent ATL‐optimized fMRI studies of healthy adult samples, however, demonstrate that the vATL is active during processing of abstract as well as concrete concepts (Bajada et al. 2019; Binney et al. 2016; Hoffman et al. 2015; Rice et al. 2018).

The present study was the first to use fMRI to examine the role of the ATL in abstract verb processing. We found an equal, positive response of the vATL to most types of verbs. We observed a less robust activation for mental state verbs relative to the baseline, yet a contrast between mental state and embodied verbs also showed no significant differences, indicating that mental state verbs do engage the vATL to some extent. Why is the vATL less engaged by abstract mental state verbs? One possibility is that there could be underlying differences in their representation which could facilitate syntactic classification and preclude a requirement for deeper semantic analysis. For example, verbs describing mental processing (e.g., *realize*, *foresee*, *ponder*) might, by virtue of being less differentiated and/or more semantically diverse, trigger rapid retrieval of a general sense of meaning that is sufficient for making the syntactic judgment, but does not necessarily require delving into specific representations subserved by the vATL. If such an account were correct, then more demanding semantic tasks (e.g., pairwise relatedness judgments) should evoke robust vATL activation for mental state verbs.

In addition to the vATL, regions in the IFG and posterior MTG, as well as the middle and inferior occipital gyri were active for all verb types, consistent with their proposed role in controlled retrieval of semantic information (Hodgson et al. 2021; Jefferies 2013; Thompson‐Schill et al. 1997; Whitney et al. 2011) and reading written words (Bonandrini et al. 2024) respectively. We also observed activity in the right cerebellum, consistent with evidence that the right cerebellum is functionally involved in syntactic and semantic processing (Nakatani et al. 2023) and reading (Alvarez and Fiez 2018; Martin et al. 2015).

### 7.2 The Graded ATL Semantic Hub

The MVPA analyses revealed several clusters of voxels over which activity can be used to classify each abstract verb type, relative to the embodied verbs. This overall pattern is consistent with a graded semantic hub account that characterizes anterior temporal cortex as a unified representational space, but one that exhibits gradients of connectivity that give rise to shifts in semantic function (Bajada et al. 2019; Binney et al. 2012; Rice et al. 2015). At the center of this space lies the ventrolateral ATL, which is engaged by all concept types and semantic information of any kind. Towards the edges, there are gradual shifts in semantic function such that regions on the periphery are relatively more specialized for (i) receiving inputs from particular modalities (e.g., vision or audition), and (ii) encoding certain types of semantic features (for a computational exploration of this general hypothesis, see Plaut 2002).

We observed that mental state verbs are associated with voxels in the bilateral temporal poles, and this could align with prior studies that attribute a role to the poles in processing social concepts (Balgova et al. 2022, 2024; Binney et al. 2016; Lin et al. 2018; Olson et al. 2007). Specifically, the mental state verbs in the present study are related to cognition and are defined as mental actions or processes of acquiring knowledge (Muraki et al. 2022). Thus, the meaning of many of these verbs could include socially relevant information, such as information about one's own and others' mental states, driving involvement of the poles.

In the present study, emotional verbs were associated with an ATL subregion located in the anterior to middle MTG of the left hemisphere. Previous studies have found that emotional semantic information drives bilateral temporal pole activation, in a similar way to social concepts, and it has been suggested that this reflects direct connectivity of the poles with limbic regions (Binney et al. 2012; Wang et al. 2019). The region identified here is more posterior and has been associated with processing more general socioemotional qualities of concepts (Hung et al. 2020; Wang et al. 2019). It is also broadly implicated in social cognition, along with the superior temporal sulcus (Deen et al. 2015; Jackson et al. 2023), and this has been linked to the MTG's role as part of a default mode network (Hung et al. 2020) that supports internally generated (perceptually‐decoupled) cognitive processes, such as mind‐wandering, self‐referential activities, autobiographical memory, and emotional processing (Raichle 2015; Satpute and Lindquist 2019). Satpute and Lindquist (2019) propose that the function of the default mode network in emotional processing is to abstract away from physiological features of emotional experience and encode the multimodal conceptualization of an emotional category.

Finally, nonembodied verbs were associated with a cluster slightly anterior to that for emotional verbs, as well as a more ventromedial cluster of voxels in the ITG. ITG involvement could reflect graded ATL functional sensitivity to visual semantic information (Hoffman et al. 2015). Given that nonembodied verbs refer to actions, states, or relations that occur external to the human body, it is possible that they are associated with more perceptual experience and this drives inputs to the inferior ATL from adjacent visual association regions. All the above possibilities will need to be explored further in future research.

### 7.3 Differential Engagement of Modality‐Specific Spokes for Different Categories of Verbs

In the present study, we compared categories of verbs that varied in the degree to which sensorimotor, emotional, and other interoceptive experience (i.e., cognition) contributes to their representation (according to ratings studies). Contrary to previous studies, we found only weak evidence of category‐specific activation in regions outside of the ATL. This manifested mainly in the extent of activation rather than regional dissociations, with the exception of left precentral gyrus and bilateral postcentral gyrus activity which appeared specific to nonembodied verbs. These findings were, however, only observed in the contrast against the numerical baseline task and were absent from direct comparison between verb types. Overall, our results do not support the hypotheses that emotional verbs are associated with activity in the bilateral amygdalae, mental state verbs are associated with activity in the left temporal parietal junction, or that nonembodied verbs are associated with activity in the left MTG.

There are several possible alternative explanations for these null findings. The first relates, once again, to the nature of the task demands, and the possibility they do not drive the semantic system hard enough to elicit robust differences between categories. Participants were asked to discriminate verbs from nouns, not to distinguish between different types of verbs, and this may have limited the depth of semantic analysis required. This would appear inconsistent, however, with the finding that all verb types engaged the vATL to some extent. A second possibility is that there is a high degree of heterogeneity among concepts within a category, and this drives high variability of engagement both across and within spoke regions, which will be lost to signal averaging. The emergence of differential downstream activity across ATL subregions (see above) is not incompatible with this, as it could reflect convergence and summation of inputs from several pathways. Finally, a third possibility is that all abstract verbs are heavily reliant on linguistic information and minimally engage sensorimotor, affective, or interoceptive neural systems. Engagement of language systems in processing abstract concepts was associated with activation of the left IFG by Hoffman and Bair (2024) in their recent large meta‐analysis of neuroimaging studies and with the pars orbitalis of the IFG and left anterior STS by Binder et al. (2009) in their meta‐analysis of neuroimaging studies. Semantic control has also been associated with the IFG. Here, we observed IFG activation in response to all abstract verb types. Abstract words also tend to occur in more diverse semantic contexts (Hoffman et al. 2013) and require a greater degree of cognitive control to ensure the correct meaning is retrieved (Hoffman et al. 2015; Hoffman and Bair 2024). However, these explanations would appear somewhat incompatible with another of our findings. Contrary to our expectations that the nonembodied verbs would be the most abstract and therefore the most reliant on linguistic representations, they were the only verbs associated with activation of sensorimotor neural areas. One possible interpretation of this is that the nonembodied verbs evoked event representations that engage sensorimotor systems (Grush 2004; Schütz‐Bosbach and Prinz 2007). For example, many of these stimuli referred to changes of state that could *involve* the human body even if they do not *affect* the body (e.g., *uncork*, *ignite*, *tenderize*). These verbs have low embodiment ratings but may, nonetheless, include action‐related sensorimotor information as part of their meanings.

## 8 Conclusions and Future Directions

To our knowledge, the present study is the first to test the hub‐and‐spoke account in relation to abstract verb processing. Our findings extend previous research, providing novel evidence that the vATL is engaged in embodied and nonembodied verb processing, adding to the list of concept types that appear to be represented by the vATL. Further, we found evidence to support the graded semantic hub hypothesis, namely differential activity of ATL subregions for different types of verbs. We found little support for strong modality‐specificity associated with the verbs, in that we were unable to obtain evidence for differential activity in distributed modality‐specific regions. Although the verb stimuli were carefully selected, the categories of abstract verbs used here could fail to adequately capture heterogeneity in the recruitment of modality‐specific systems. This may suggest that abstract verbs draw upon multiple, overlapping sources of information and experience. Future research could explore other direct tests of the hub‐and‐spoke model. One option may be to assess functional connectivity of the hub and spokes for various types of abstract verbs using a graph‐theory network analysis approach. Or, to examine representations within the hub‐and‐spoke model, we propose the use of representational similarity analyses to correlate patterns of brain activity with a richer and continuous set of information about the dimensions underlying abstract word meaning.

## Author Contributions

**Emiko J. Muraki:** conceptualization, data curation, formal analysis, funding acquisition, investigation, methodology, resources, visualization, writing – original draft, writing – review and editing. **Penny M. Pexman:** conceptualization, funding acquisition, supervision, writing – review and editing. **Richard J. Binney:** conceptualization, data curation, funding acquisition, investigation, methodology, resources, supervision, writing – review and editing.

## Conflicts of Interest

The authors declare no conflicts of interest.

## Supporting information


Data S1.

## Data Availability

The data that support the findings of this study are openly available in OSF at https://osf.io/vd9ba/.

## Appendix 1 Verb Stimuli

**Table:**

Embodied	Emotional	Mental	Nonembodied
Apprehend	Annihilate	Acquaint	Accelerate
Breastfeed	Backfire	Analyse	Besiege
Collide	Banish	Assert	Broaden
Devour	Bombard	Assess	Cater
Dilate	Capsize	Attest	Congregate
Dislodge	Colonize	Authorise	Contain
Dismount	Confiscate	Calculate	Decelerate
Disrobe	Decompose	Coerce	Dismantle
Distribute	Deport	Compute	Dispense
Eavesdrop	Derail	Conclude	Embark
Excrete	Desecrate	Condone	Embed
Extend	Detonate	Contrive	Encircle
Fornicate	Devastate	Convey	Enlarge
Gawk	Discard	Decode	Escalate
Grapple	Disrupt	Deduce	Excavate
Hasten	Erode	Define	Exhume
Hoist	Evict	Devise	Expend
Immigrate	Expire	Discern	Ferment
Incubate	Fester	Equate	Ignite
Ingest	Flunk	Evaluate	Insulate
Inhabit	Hinder	Exemplify	Invert
Insert	Implode	Foretell	Magnetize
Linger	Impoverish	Formalize	Occur
Marinate	Infest	Generalize	Originate
Metabolize	Misfire	Idealize	Outlast
Migrate	Mooch	Imply	Propel
Mutter	Negate	Initiate	Reduce
Outrun	Overburden	Inquire	Regroup
Outweigh	Overload	Introspect	Reside
Populate	Overshadow	Outwit	Resurface
Quench	Overstep	Predict	Revert
Recoil	Perish	Reassign	Saturate
Salivate	Pollute	Reconsider	Solidify
Scuttle	Regress	Repent	Surpass
Secrete	Remove	Restate	Tenderize
Straddle	Skimp	Revise	Transfuse
Unpack	Squander	Specify	Uncork
Urinate	Stagnate	Stipulate	Unleash
Vaccinate	Underpay	Supervise	Unveil
Vocalise	Wither	Symbolize	Vanquish

## Appendix 2 Noun Stimuli

**Table:**

High BOI		Low BOI	
Applicator	Longbow	Abrasion	Migration
Avocado	Mallet	Abyss	Mileage
Badminton	Manicurist	Airflow	Monogram
Banknote	Marmalade	Allergy	Novice
Beaker	Mascara	Analogy	Overpass
Blazer	Megaphone	Apostrophe	Overture
Bourbon	Mousse	Bedrock	Parasite
Caviar	Mutton	Centaur	Particle
Converter	Opium	Cherub	Periwinkle
Corduroy	Oregano	Chromosome	Quantity
Corset	Peppercorn	Convulsion	Quota
Crossbar	Quiche	Corrosion	Rarity
Crossbow	Ringworm	Diameter	Refinery
Curbside	Sapphire	Elevation	Regime
Cutlery	Saxophone	Emblem	Sanitation
Dashboard	Slipknot	Encore	Scribe
Decor	Soybean	Esquire	Sealer
Dipstick	Stockroom	Famine	Seascape
Dumpling	Stockyard	Fraction	Sideshow
Enamel	Strudel	Gargoyle	Siege
Forklift	Suede	Hygiene	Skit
Gecko	Sunroof	Hyphen	Slogan
Goblet	Surveyor	Indigo	Sunburst
Hearth	Terrier	Irritant	Sunstroke
Hostess	Thesaurus	Kilogram	Tempest
Javelin	Thyme	Kilowatt	Turbulence
Jeweler	Toupee	Malice	Typhoon
Joystick	Turban	Margin	Vowel
Lantern	Villa	Memoir	Waiver
Logbook	Watercress	Methane	Warhorse
