# Supplementary material for: Mapping Contributions of the Anterior Temporal Semantic Hub to the Processing of Abstract and Concrete Verbs
Source: Hum Brain Mapp. 2025 Apr 24;46(6):e70210. doi: 10.1002/hbm.70210 (PMC12021998; doi:10.1002/hbm.70210)
Supplement: Supplementary file 1 — Data S1. [file HBM-46-e70210-s001.docx]

**Supplemental Materials:**

**Mapping Contributions of the Anterior Temporal Semantic Hub to the Processing of Abstract and Concrete Verbs**

Emiko J. Muraki^1,2^, Penny M. Pexman^1,2,3^, and Richard J. Binney^4^

^1^ Department of Psychology, University of Calgary

^2^ Hotchkiss Brain Institute, University of Calgary

^3^ Department of Psychology, Western University

^4^ Cognitive Neuroscience Institute, School of Psychology and Sport Science, Bangor University

**Supplemental Table 1**

*Descriptive Statistics for Word Stimuli*

| Word Type  (n = 40 per verb type  n = 60 per noun type) | Emb. M  SD | Valence M  SD | Cog. M  SD | Freq M  SD | Length M  SD | AoA M  SD | BOI M  SD |
| --- | --- | --- | --- | --- | --- | --- | --- |
| Mental abstract verbs | 2.86  0.55 | 5.28  0.62 | 5.59^c^  0.39 | 1.58  0.52 | 7.38  1.33 | 10.85  1.36 | na |
| Emotional abstract verbs | 3.12  0.52 | 2.94^b^  0.62 | 2.65  0.49 | 1.43  0.52 | 7.20  1.56 | 10.68  1.58 | na |
| Nonembodied abstract verbs | 3.11  0.60 | 5.29  0.70 | 2.67  0.42 | 1.42  0.51 | 7.30  1.42 | 10.84  1.48 | na |
| Embodied verbs | 5.20^a^  0.80 | 5.12  0.58 | 2.48  0.55 | 1.43  0.45 | 7.28  1.41 | 10.57  1.47 | na |
| High BOI nouns | na | na | na | 1.50  0.41 | 7.30  1.41 | 10.71  1.29 | 5.13^d^  0.62 |
| Low BOI nouns | na | na | na | 1.52  0.43 | 7.28  1.45 | 10.71  1.35 | 2.27  0.49 |

Note. Ratings included are embodiment (Emb.), valence, cognition (Cog.), frequency (Freq.), Length, age of acquisition (AoA), and body-object interaction (BOI). M = Mean, SD = Standard deviation. ^a^ indicates that the embodiment ratings for the embodied verbs are significantly higher than all other verbs (p < .05). ^b^ indicates that the valence ratings for the emotional abstract verbs are significantly lower than for all other verbs (p < .05). ^c^ indicates that the cognitive ratings for the cognitive abstract verbs are significantly higher than for all other verbs (p < .05).^d^ indicates that the BOI ratings for the high BOI nouns are significantly higher than for the low BOI nouns (p < .05). There are no significant differences between any of the stimuli groups on length, frequency, or age of acquisition

**Supplemental Table 2**

*Mixed Effects Models Predicting SCT Response Times and Accuracy*

|  | Linear Regression - Response Times | | | Logistic Regression - Accuracy | | |
| --- | --- | --- | --- | --- | --- | --- |
| Fixed Effects | b | 95% CI | p | OR | 95% CI | p |
| Intercept | 900.66 | [838.70, 962.63] | < .001 | 21.20 | [11.86, 37.88] | < .001 |
| Verb Type (Emotional) | 13.94 | [-17.69, 45.58] | .388 | 0.67 | [0.45, 1.01] | .056 |
| Verb Type (Mental) | -9.23 | [-40.70, 22.23] | .565 | 1.25 | [0.82, 1.92] | .298 |
| Verb Type (Nonembodied) | 9.72 | [-21.68, 41.12] | .544 | 0.79 | [0.52, 1.18] | .249 |
| Random Effects | Variance | SD |  | Variance | SD |  |
| Participant Intercept | 24388 | 156.17 |  | 0.36 | 0.60 |  |
| Item Intercept | 3168 | 56.28 |  | 1.71 | 1.31 |  |
| Residual | 48484 | 220.19 |  |  |  |  |
| Model Fit | Marginal | Conditional |  | Marginal | Conditional |  |
| R^2^ | .001 | .363 |  | .010 | .393 |  |

Note. CI = confidence interval; OR = odds ratio; Verb type is a factor variable with embodied verbs set as the reference group. Accuracy is a binary dependent variable with inaccurate responses as the reference group (0) and accurate responses as the focus group (1). The marginal R^2^ includes only the variance from the fixed effects and the conditional R^2^ includes variance from both the fixed and random effects. The model equations for response time and accuracy respectively were: RT ~ Verb Type + (1|Participant) + (1|Item) and Acc ~ Verb Type + (1|Participant) + (1|Item). p-values for fixed effects in the linear mixed effects model are calculated using Satterthwaite's method. N participants = 28, N stimuli = 158.

**Supplemental Table 3**

*Mixed Effects Models Predicting Task Response Times and Accuracy*

|  | Linear Regression - Response Times | | | Logistic Regression - Accuracy | | |
| --- | --- | --- | --- | --- | --- | --- |
| Fixed Effects | b | 95% CI | p | OR | 95% CI | p |
| Intercept | 800.17 | [751.29, 849.05] | **< .001** | 29.66 | [19.51, 45.09] | **< .001** |
| Task (SCT) | 116.87 | [101.32, 132.41] | **< .001** | 0.63 | [0.48, 0.82] | **.001** |
| Random Effects | Variance | SD |  | Variance | SD |  |
| Participant intercept | 16206 | 58.01 |  | 0.87 | 0.93 |  |
| Item Intercept | 3365 | 127.30 |  | 0.91 | 0.96 |  |
| Residual | 53645 | 231.61 |  |  |  |  |
| Model Fit | Marginal | Conditional |  | Marginal | Conditional |  |
| R^2^ | .039 | .296 |  | .009 | .358 |  |

Note. CI = confidence interval; OR = odds ratio; Task is a binary variable with NJT set as the reference group. Accuracy is a binary dependent variable with inaccurate responses as the reference group (0) and accurate responses as the focus group (1). The marginal R^2^ includes only the variance from the fixed effects and the conditional R^2^ includes variance from both the fixed and random effects. The model equations for response time and accuracy respectively were RT ~ Task + (1|Participant) + (1|Item) and Acc ~ Task + (1|Participant) + (1|Item). p-values for fixed effects in the linear mixed effects model are calculated using Satterthwaite's method. N participants = 28, N stimuli = 406.
